# Digital Youth and Family Engagement Program for Adolescents Who Receive Outpatient Mental Health Services: Qualitative Evaluation

**DOI:** 10.2196/60317

**Published:** 2024-10-31

**Authors:** Ana Ramirez, Justin Kramer, Katrina Hazim, Jason Roberge

**Affiliations:** 1 Department of Anthropology University of North Carolina at Chapel Hill Chapel Hill, NC United States; 2 Center for Health System Sciences Atrium Health Charlotte, NC United States; 3 Department of Family and Community Medicine Wake Forest University School of Medicine Winston-Salem, NC United States; 4 Carolinas Medical Center Atrium Health Charlotte, NC United States

**Keywords:** telemedicine, telepsychiatry, adolescents, mental health, psychiatry, coaching, qualitative assessment, patient satisfaction, family engagement, depression, anxiety, suicidal ideation

## Abstract

**Background:**

Incidents of depression, anxiety, and suicidal ideation among adolescents have increased in recent years. Mental health interventions tailored to adolescents and families need to consider mechanisms for increasing enrollment and sustaining program engagement. A telephone-based, health coach intervention for adolescents and families was implemented at a Southeastern US health system with the goals of improving psychiatric appointment attendance, medication adherence, reduction in emergency department visits, and assisting with crisis management (“Youth and Family Engagement” [YFE] program).

**Objective:**

This study aims to explore patients’ and parents’ perceptions of a mental health program and the factors that impact enrollment and sustained engagement.

**Methods:**

Semistructured interviews were conducted with adolescent patients (n=9, 56%), parents (n=11, 92%), and clinicians who placed patient referrals (n=6, 100%). Interviews were in English (participants: 19/26, 73%) or Spanish (parents: 7/11, 64%), depending upon participants’ preference. Interviews explored perceptions of the YFE program, experiences working with health coaches, suggestions for program changes, and program goals. The data were analyzed using inductive coding methodologies, with thematic analysis used to organize emergent themes. Two qualitatively trained researchers, one bilingual in English and Spanish, facilitated all data collection and collaboratively performed data analysis.

**Results:**

The YFE program’s structure was often mentioned as promoting engagement, with telephone appointments and health coaches’ ability to accommodate inflexible work or school schedules alleviating participation barriers. Skills learned from health coaches were frequently referenced, with adolescents generally citing internal processes, such as positive thinking and mindfulness. Parents discussed behaviors relative to their children, such as improvements with discipline, setting boundaries, and improved parent-child communication. Many participants discussed the importance of health coaches assisting families in navigating social systems, such as accessing resources (eg, housing) and navigating school processes (eg, individualized educational plans), with clinicians suggesting an increased emphasis on adolescents’ nutrition and engagement in primary care. Spanish-speaking parents highlighted numerous advantages of working with bilingual health coaches, emphasizing both enhanced communication and cultural understanding. They specifically noted the coaches’ ability to grasp their lived experiences and challenges as immigrants in the United States, which significantly enriched their participation in the program.

**Conclusions:**

Prioritizing convenient engagement for adolescents and families may be important for sustained program participation, as inflexible schedules and competing priorities pose barriers to traditional appointments. Future programs should carefully consider health coach–participant relationships, specifically cultural competency, providing services in native languages, and assisting families with wraparound care, as these may be crucial to sustained engagement.

## Introduction

There is clear evidence of a growing mental health crisis among youth aged 10-24 years. Suicide rates among this age group increased to 52.2% between 2000 and 2021, making it the second leading cause of death among this age group and accounting for 15% of all suicides [1]. Even prior to the COVID-19 pandemic, studies noted a significant increase in anxiety and depression, with a 27% and 24% increase in anxiety and depression, respectively, between 2016 and 2019 [2]. The COVID-19 pandemic further exacerbated this crisis, with evidence of increased difficulties with schoolwork, emotional abuse, physical abuse, and food insecurity among youth [3]. Pooled estimates during the pandemic suggest a doubling of clinically elevated anxiety and depression symptoms among youth [2], along with a rise in eating disorders and self-injurious behaviors [4,5].

Various digital mental health interventions have been developed to address the growing mental health needs of adolescents [6,7]. As digital connectivity continues to expand, digital engagement with mental health services provides multiple benefits, such as improved accessibility, cost-effectiveness, increased flexibility, and reduced stigma for patients [8,9]. There is evidence that youth exhibit a higher level of comfort and satisfaction with receiving treatment through technology platforms when compared to previous generations, including their parents [9,10]. Studies have shown that clinicians are able to establish similar rapport and therapeutic alliance with youth and their families through digital mental health services, due to increased client vulnerability when behind a screen [11,12]. One study even suggested the possibility of developing stronger rapport digitally due to clients feeling more comfortable and vulnerable when behind a screen [11]. Digital delivery of mental health services also allows for greater connectivity to patients’ families, as clinicians are able to see patients in their home environments, which is particularly important in the youth population [11].

Using a web-based mental health coach is one of the various forms of digital mental health interventions that have been developed [13,14]. A health coach is a mental health professional who partners with patients and families to identify their health goals and assists in developing plans for action. Through regular check-ins, a health coach discusses changes patients would like to make and supports patients in both setting goals and identifying barriers that may impact their success. Health coaches may use psychological techniques like motivational interviewing or cognitive behavioral therapy when coaching [15,16]. Mental health coaching offers accountability and support while self-pacing and flexibility allow individual needs to be met [14,17]. Patients appreciate the comfort and convenience of web- or telephone-based mental health interventions, and although technical issues can be common, they are often manageable [14,17]. Several studies have demonstrated the effectiveness of digital mental health coaching in improving mental health symptoms and overall quality of life [13,18,19]. Whereas most of the current literature on digital mental health coaching focuses on adult populations [13,14,17-19], this qualitative study evaluates a telephone-based mental health coaching intervention for adolescents and their families.

We use qualitative methodologies to assess the effectiveness of the Youth and Family Engagement (YFE) program, specifically highlighting the factors that impact participant enrollment, sustained engagement, and patients’ and parents’ overarching experiences in the program. Building upon the limited previous qualitative research exploring digital mental health interventions for adolescents [17,20], this study captures patient, parent, and clinician feedback. Exploring the facilitators and barriers to program enrollment and engagement, this study also examines cultural and language barriers, an aspect that has received limited attention thus far in the literature on digital mental health interventions [17].

## Methods

### Intervention

The YFE program is a telephone-based, health coach intervention tailored to meet the needs of adolescents and their families who need additional help (eg, access to food and housing) or adolescent patients who could benefit from case management. The program goals include improving adolescent patients’ psychiatric appointment attendance and medication adherence while reducing emergency department visits and assisting with crisis management (eg, suicidal ideation). The YFE program was implemented in the winter of 2022 at a large, academic learning health system in the Southeastern United States. All adolescent participants in the YFE program receive mental health services through the academic learning health system regularly meeting with a counselor, therapist, or psychiatrist, which can occur in either outpatient or school-based settings. YFE referrals are placed by patients’ clinicians via the electronic health record, with YFE staff enrolling interested patients and families. Once in the YFE program, patients independently meet with health coaches biweekly, while parents have monthly meetings, which occur for the duration of the 6-month program. One of the 2 health coaches is bilingual, and services are in either Spanish or English, depending upon the preferences of patients and families.

### Participants

All participants were referred by YFE program staff, with the project team conducting recruitment outreach to explain the purpose and scope of the project and to schedule interviews. Patients and parents were interviewed separately. From July 2023 to October 2023, we conducted semistructured telephone interviews with adolescent patients (n=9, 56%) and parents (n=11, 92%) who participated in the YFE program, as well as video interviews (Microsoft Teams) with clinicians who placed program referrals (n=6, 100%).

### Ethical Considerations

This project was approved and deemed quality improvement by the Wake Forest University School of Medicine Institutional Review Board (IRB00088503). We followed the Consolidated Criteria for Reporting Qualitative Research (COREQ; Multimedia Appendix 1) [21]. All participants provided verbal consent prior to being interviewed. To protect the privacy of research participants, all study-related data were deidentified and given a corresponding identifier. No compensation was offered to research participants.

### Data Collection

Of those referred, semistructured interviews were completed with 9 (56%) out of 16 patients, 11 (92%) out of 12 parents, and 6 (100%) out of 6 clinicians. While we were successful in interviewing most of the individuals referred, scheduling conflicts and an inability to connect by telephone hindered the recruitment of some potential participants, particularly among adolescent patients. Interviews lasted 15-30 minutes, with all patient and clinician interviews being conducted in English, while 7 (64%) out of 11 parent interviews were in Spanish. Semistructured interview guides were collaboratively developed by the team (Multimedia Appendices 2-5), which included researchers trained in qualitative methods and who had expertise in adolescent mental health. Interview guides explored participants’ perceptions of the YFE program, experiences working with health coaches, suggestions for program changes, and program goals. Demographic characteristics were obtained from the electronic health record and via preinterview questionnaires to provide descriptive information about participants (Table 1). Two researchers with graduate-level training in qualitative methodologies, 1 bilingual female and 1 male, performed all data collection and analysis (AR and JK).

**Table 1:** Participant demographics and characteristics^a^.

				Adolescent patients (n=9)		Parents (n=11)		Referring clinicians (n=6)
**Language for interview, n (%)**								
	English			9 (100)		4 (36)		6 (100)
	Spanish			0 (0)		7 (64)		0 (0)
**Age (years), mean (SD; range)**				13.4 (2.4; 11-18)		39.5 (6.0; 31-48)		N/A^b^
**Years in practice, mean (SD; range)**				—^c^		—		18.8 (5.12; 10-21)
**Sex, n (%)**								
	Female			5 (56)		9 (82)		6 (100)
	Male			4 (44)		2 (18)		0 (0)
**Race, n (%)**								
	American Indian or Alaskan Native			1 (11)		0 (0)		1 (17)
	Asian			0 (0)		0 (0)		2 (33)
	Black or African American			3 (33)		2 (18)		0 (0)
	White			3 (33)		2 (18)		3 (50)
	Other			0 (0)		6 (55)		0 (0)
	Two or more races			0 (0)		1 (9)		0 (0)
	Declined			2 (22)		0 (0)		0 (0)
**Ethnicity, n (%)**								
	Hispanic			4 (44)		8 (73)		0 (0)
	Non-Hispanic			4 (44)		3 (27)		6 (100)
	Not reported			1 (12)		0 (0)		0 (0)
**Adolescents’ mental health services, n (%)**								
	**Counseling**							
		In-school only	4 (44)		2 (18)		—	
		External to school only	4 (44)		7 (64)		—	
		Both in-school and external	1 (12)		2 (18)		—	
	**Medication management**							
		In-school only	0 (0)		0 (0)		—	
		External to school only	0 (0)		4 (36)		—	
		None	9 (100)		7 (64)		—	
**Self-reported health, n (%)**								
	Excellent			4 (44)		0 (0)		—
	Good			3 (33)		6 (55)		—
	Fair			2 (22)		4 (36)		—
	Poor			0 (0)		1 (9)		—
**Mental health position, n (%)**								
	Outpatient psychiatrist			—		—		3 (50)
	School-based therapist			—		—		3 (50)
**Education, n (%)**								
	Less than high school			—		3 (27)		—
	High school diploma			—		4 (36)		—
	Some college			—		2 (18)		—
	Associates degree			—		1 (9)		—
	Declined			—		1 (9)		—
**Employment, n (%)**								
	Full time			—		7 (64)		—
	Part time			—		2 (18)		—
	Not in labor force			—		2 (18)		—
	Unemployed			—		0 (0)		—
**Living situation, n (%)**								
	Steady place			—		7 (64)		—
	Steady today, but worried			—		4 (36)		—
	No steady place			—		0 (0)		—

^a^From July 2023 to October 2023, semistructured interviews were conducted with adolescent patients (9/16, 56%), parents (11/12, 92%), and clinicians (6/6, 100%) to capture feedback on the Youth and Family Engagement program.

^b^N/A: not available.

^c^Not applicable.

### Analysis

Semistructured interviews (N=26) were audio recorded, transcribed verbatim, deidentified, and analyzed using ATLAS.ti software (ATLAS.ti Scientific Software Development GmbH). Inductive coding methodologies were used to capture emergent themes in the data, with thematic analysis strategies used to systematically organize data [22]. The project team collaboratively developed a codebook, which included 8 parent codes and 20 subcodes, that AR or JK then used to independently code the same 3 transcripts (>10%). Any discrepancies were reconciled, and the codebook was refined as necessary, with the remaining transcripts being independently coded by either AR or JK. Questions that arose during the analysis were presented to the wider team for review. Our sample size was sufficient to reach thematic saturation. Participants did not assist with data analysis, interpretation of findings, or transcription verification processes, as these were handled by the study team.

## Results

### Sample

Interviews were conducted with 26 participants: 9 (56%) out of 16 patients, 11 (92%) out of 12 parents, and 6 (100%) out of 6 clinicians. All patient and clinician interviews were conducted in English, while most parent interviews (7/11, 64%) were conducted in Spanish. Patients were demographically diverse across sex (female: 5/9, 56%; male: 4/9, 44%), race (Black or African American: 3/9, 33%; White: 3/9, 33%; and American Indian or Alaskan Native: 1/9, 11%), and ethnicity (Hispanic: 4/9, 44% and non-Hispanic: 4/9, 44%), with an age range of between 11 and 18 (mean 13.4, SD 2.4) years. Participating parents were mostly female (9/11, 82%) and Hispanic (8/11, 73%), with an age range between 31 and 48 (mean 39.5, SD 6.0) years. All clinician participants were non-Hispanic and female; however, they were diverse across race (White: 3/6, 50%; Asian: 2/6, 33%; and American Indian or Alaskan Native: 1/6, 17%); clinical role (psychiatrists: 3/6, 50%; therapists: 3/6, 50%); and years of clinical practice, which ranged from 10 to 21 (mean 18.8, SD 5.12) years. Further demographic data can be found in Table 1.

### Themes

#### Overview

During analysis, several themes emerged about participants’ experiences in the YFE program, including (1) the convenience of digital appointments, (2) the importance of health coaches providing effective and tailored support to meet participants’ needs, (3) the capacity of health coaches to teach participants key mental health skills, and (4) the benefits associated with partnering Spanish-speaking participants with bilingual health coaches (Table 2). Further, we identified suggestions for the expansion and improvement of our digital mental health program with health coaches (Table 3).

**Table 2:** Perceptions of the YFE^a^ program: themes and illustrative quotes. Using inductive coding methodologies to capture emergent themes in the data, 4 themes were identified in the semistructured interviews with participants, along with accompanying quotes.

Themes	Illustrative quotes
Theme 1: Convenience of digital appointments (eg, scheduling, telephone, health coaches)	“I think when most people think of therapy, they think of having to drive up to a place and sit there, but really, like I said, the great thing about it is just having the calls and being able to call back. Really, the main pro of it is just convenience.” [Patient, non-Hispanic White, male] “The fact that it is available over the phone not that I'm so busy, but it’s just not always a good time for me to be available to come to an appointment so that’s really appreciated. It works well for both of us.” [Mother, non-Hispanic Black, aged 47 years] “I think that YFE, having that shifted schedule—because I think they make calls until 7:00 or something like that. I think that’s so amazing, especially for this mom that I’m thinking of. She can’t always answer the phone during work. A lot of times, it’s after work. I think just that flexibility that the YFE Program provides that I can’t necessarily provide as a therapist.” [Therapist, non-Hispanic White, female]
Theme 2: Coaching provides tailored support to effectively address participants’ needs.	“My son was going to school since he was seven in a special education school. Then when I got here, they don’t have those types of programs...That’s why I wanted to have more information, and that I can have somebody if I got any doubts, they can guide me and tell me if it is anything else out there that I can do to help him.” [Mother, Hispanic, aged 37 years] “She [the health coach] started the process of giving me names to find a place like a shelter...That’s how I got into Charlotte Family Housing. On top of it, she helped me to get food and stuff like that, so yes. She’s been my friend. She’s my angel.” [Mother, non-Hispanic Black, aged 46 years] “Yeah, the parent[s], they love talking to [the health coach]. They love—they talk about the support. It’s a nice bridge, yes. I think it’s been a huge resource. As far as what I heard from the parent, it’s been a massive resource for them.” [Therapist, non-Hispanic White, female]
Theme 3: Health coaching equips participants with key mental health skills.	“Yes, she says to spend more time together, if she wants to go to the gym to let her go, cook together, watch a TV show together, go for walks. All that would help our father/daughter relationship...everything she said we should do, it all worked.” [Father, Hispanic, aged 36 years, Spanish-speaking] “Yeah, so my health coach, really, we like to emphasize positive thinking and doing what you can with what you have...Look at the variables that you can control, and fixing those, but the things that you cannot control, not worrying about them.” [Patient, non-Hispanic, male, aged 16 years] “Well, the first goal was when we had—because when we started going to Atrium because of her suicidal attempt and stuff, it was basically parent and child conflict...When [the health coach] started calling me, she was telling me what to say to her.” [Mother, non-Hispanic Black, aged 46 years]
Theme 4: Spanish-speaking participants value bilingual health coaches	“We’re all alone in this country and I find the support that you provide super useful. All the emotional support; the fact that they care about them. I think that’s super useful.” [Mother, Hispanic, 35, Spanish-speaking] “Thankfully, we did not need an interpreter because she speaks my language. She tells me that if I need anything translated, she can do that for me, too. I think that’s why I like her so much.” [Mother, Hispanic, aged 43 years, Spanish-speaking]

^a^YFE: Youth and Family Engagement.

**Table 3:** Suggestions for YFE^a^ program changes. Thematic analysis of semistructured interview content helped identify 5 suggestions for YFE program changes.

Suggestions	Illustrative quotes
Suggestion 1: Video and FaceTime expansion for health coach meetings	“The only thing I could think of would be home visits, because in the two cases, one was disabled. Both of them did not have transportation. I know it’s just on the phone, but they’re people that have difficulty connecting and establishing rapport, so I think a face-to-face type situation where someone went to them might be good.” [Licensed social worker, non-Hispanic White, female] “[Health Coach] told me that the next call they were going to use a video call, through Zoom and that is very good, honestly. Because you interact much better like this. To be in a video call and know who you are talking to.” [Father, Hispanic, aged 34 years, Spanish-speaking] “I would usually prefer in-person, but Zoom could probably work too...I’m usually used to in-person since I used to always do it in person. Normally now I just do virtual just like phone...People would probably mostly like in-person because you wouldn’t know their face, and you would probably want to know them.” [Patient, non-Hispanic White, female, aged 13 years]
Suggestion 2: Flexible program duration and health coach meeting intervals	“[LONGER] It really depends on the issue that you’re having, but I’m just unsure about the time limit [6 months], I guess. I feel like it could be longer. Some people probably need longer than that to talk when they’re getting out of a rough spot, and some people may not need that much. I think just putting a timestamp on that isn’t really the best way.” [Patient, non-Hispanic White, male, aged 16 years] “[SHORTER] I feel like shorter, maybe a couple months but I feel like six months is a lot.” [Patient, female, aged 14 years]
Suggestion 3: Expanded program content (eg, bullying, nutrition)	“Something that helps me with that bullying thing so it’s not the same this year...I would say that providing better opinions to my son...Because of all that he’s been going through. To make it different; giving him advice.” [Mother, non-Hispanic White, aged 31 years] “If there was more health coaching in terms of diet and nutrition, along those lines, and just managing the health of kids, that would be important...People with lower socio-economic status have higher risk of childhood obesity and stuff like that.” [Psychiatrist, non-Hispanic American Indian, female] “Any type of liaisoning with schools, because a lot of these kids have trouble both at home and at school. A lot of the stress and trouble seems to happen with school. I don’t know how much they help the families navigate some of that...More behavioral. The behavioral, emotional concerns that arise with school.” [Psychiatrist, non-Hispanic Asian, female]
Suggestion 4: Increased emphasis on connecting patients with mental health care and PCPs^b^	“Expanding it to make sure, ‘hey, are they getting primary care checkups,’ and really that case management through a lens of mental health. I just think that’s really cool.” [Therapist, non-Hispanic White, female] “There’s one I’d say probably has some medical issues. They need to make sure they’re getting treatment and following up with their primary care doctor.” [Psychiatrist, non-Hispanic American Indian, female] “With the program, what exactly are they hoping to accomplish from this? If it is to get connected with therapy, I think they need a different referral-coordination process because I don't think that that’s happened with some patients.” [Psychiatrist, non-Hispanic Asian, female]
Suggestion 5: Recruitment efforts to include other school officials or leaders and target schools with lower SES^c^ student populations	“I’m really excited for them to expand to getting referrals from the school...The school counsellors obviously can’t see their notes, but I just think in terms of connecting with the community and getting more people into these types of things. That’s so helpful. My hope would be that type of thing can help get some of those kids before they fall through the cracks. I’m excited for the growth and expansion and everything it can do.” [Therapist, non-Hispanic White, female] “My other school is certainly—it’s a Title 1, low-income school, and I think there’s more of a need there...The families are a little more high-need, that I’m thinking more of how the YFE Program could be beneficial if the parents on board and the kids on board.” [Therapist, non-Hispanic White, female]

^a^YFE: Youth and Family Engagement.

^b^PCP: primary care providers.

^c^SES: socioeconomic status.

#### Theme 1: Convenience of Digital Appointments

An aspect of the YFE program that patients, parents, and clinicians repeatedly pointed out is the convenience of digital appointments (eg, scheduling and access to health coaches). Both parents and adolescent patients expressed value in the ease and flexibility of reaching health coaches through telephone calls rather than in-person sessions. For example, several parents explained that their work and life schedules can complicate attending in-person sessions; therefore, the digital appointments in the YFE program made their participation easier and possible. Patients similarly valued the flexibility of calls with health coaches, as many expressed the need to balance school schedules and extracurricular activities. Additionally, 1 clinician noted that the program’s flexible schedule allowed health coaches to meet with participants after regular working hours, which removed a common barrier to program engagement, particularly for parents with inflexible work schedules. Overall, participants felt that the digital aspect of the YFE program provided convenient and accessible mental health support for both parents and adolescent patients.

#### Theme 2: Coaching Provides Tailored Support to Meet Participants’ Needs

In the YFE program, health coaches provide one-on-one tailored support to participants. Both parents and patients expressed that they found it valuable and useful to meet with their health coaches at the beginning of the program to identify goals, with adolescent patients, parents, and health coaches all participating in these discussions. This tailored support was reported to make sessions useful and allow participants to work toward their goals. For example, a Hispanic mother in the program shared that her main goal was to obtain assistance navigating her son’s special needs at school. She had recently moved to the area and was unfamiliar with how to help her son in the new school setting. In her case, the health coach was able to provide more information and direct her to the necessary resources. Other parents in the program recounted receiving similar support from their health coaches, who aided them in securing resources, such as access to shelter or food, and acted as advocates. One parent even described her health coach as her “friend” and her “angel.” Clinician participants also noted that health coaches’ tailored support helps bridge the gap between families and community resources, serving as a massive source of support for families.

#### Theme 3: Coaching Equips Participants With Key Mental Health Skills

Parents commonly reported perceptions that health coaching strengthens familial bonds between parent and child, with many noting that they observed improvements in their relationships after incorporating the skills or suggestions offered by health coaches. A unique aspect of the YFE program is that health coaches work with both parents and adolescents, meeting with them separately and working on interrelated goals. Patients expressed that skills such as positive thinking and proactive problem-solving help them focus on actionable steps within their control while letting go of stressors beyond their influence. Many parents attributed their improved parent-child communication to the personalized guidance provided by health coaches, which parents felt equipped them with strategies to navigate conflicts and foster understanding with their children. Several parents explained that their health coaches encouraged them to find shared activities to do with their child to build their relationship, such as cooking, exercising, or doing an activity outside. One Hispanic father in the program recounted that he started spending more time with his daughter by going to the gym, cooking, and going on walks together and that “it all worked.”

#### Theme 4: Spanish-Speaking Participants Value Bilingual Health Coaches

Spanish-speaking parents expressed value in the emotional support, enhanced communication, and cultural understanding that came from working with bilingual health coaches. One Hispanic mother highlighted the significance of feeling supported and not alone in a foreign country, emphasizing the usefulness of such bilingual support. Another mother appreciated the ability to communicate directly with her health coach in her native language, eliminating the need for an interpreter and fostering a deeper connection.

### Suggestions for Change

#### Overview

In analyzing our data, several valuable insights and suggestions emerged for enhancing program effectiveness, such as (1) expanding health coach meetings to include video and FaceTime (Apple Inc), (2) flexible program duration and health coach meeting intervals, (3) expanded program content, (4) increased emphasis on connecting patients with PCPs and mental health care, and (5) expanding recruitment efforts to target schools with lower socioeconomic status (SES). Drawing from these key suggestions and quotes, Table 3 shares actionable recommendations that may benefit similar programs.

#### Suggestion 1: Video and Facetime Expansion for Health Coach Meetings

Clinicians, parents, and patients all mentioned the possible benefits of expanding health coach meetings beyond telephone calls and leveraging either in-person or video sessions. One parent remarked that incorporating video calls with health coaches would be “very good,” since it would enable participants to see who they “are talking to.” Similarly, other participants noted that including in-person or video sessions could be impactful for developing improved relationships with health coaches, as they felt that might be more personal than telephone calls.

#### Suggestion 2: Flexible Program Duration and Health Coach Meeting Intervals

Participants detailed engaging their health coaches to address varying needs, with some focusing on exercises to better control their emotions and others addressing urgent issues like finding stable housing or helping their child through suicidal ideation. Parents often noted that the differing needs of their children could be better met by augmenting health coach meeting intervals, either more or less frequent, depending upon current needs. For example, 1 parent observed that her child is going through more peer pressure at school, so meeting more frequently with her health coach could help her child at school. Parents also cited the differing communication styles of their children when referencing meeting intervals, with 1 parent reporting that their child prefers not to engage in frequent communication, so they suggested that meeting less frequently would be more beneficial. The overall duration of the YFE program was mentioned by a couple of patients, specifically insofar as different circumstances or life challenges (eg, social anxiety) might necessitate a longer period of support from health coaches.

#### Suggestion 3: Expanded Program Content

Parents and clinicians recommended expanding the content of the YFE program to include topics such as bullying, nutrition, and school-related stressors. Additionally, while the YFE program was designed to focus on supporting the behavioral health of adolescent patients alongside their parents, several parents reported that having a space to candidly discuss their parenting experiences was beneficial and increased their confidence in parenting an adolescent child.

#### Suggestion 4: Increased Emphasis on Connecting Patients with Mental Health Care and PCPs

Multiple clinicians noted that the established rapport between health coaches and participants represents a significant opportunity to connect participants with primary care and mental health services, particularly given the fact that all adolescents in the YFE program are active patients within the larger health system. For example, 1 clinician noted that health coaches could facilitate participants getting primary care checkups if medical issues are discussed or could help make patient referrals if there is a need for a therapist.

#### Suggestion 5: Recruitment Efforts to Include Other School Officials or Leaders and Target Schools With Lower SES Student Populations

Clinicians offered 2 specific suggestions for increasing both the enrollment in, and impact of, the YFE program—expand in-school referral processes to involve other school officials (beyond therapists) and specifically target lower SES schools. Clinicians detailed perceptions that the YFE program is most helpful for families navigating multiple social stressors and resource limitations (eg, migration, language barriers, special needs support, unstable housing) and that overall program impact, and potentially recruitment numbers, could be improved by targeting schools that might be differentially impacted by these social conditions.

## Discussion

### Principal Findings

This study contributes to the literature on telehealth programs, specifically related to mental health, by qualitatively examining key themes that impact patient experiences. Results from this study may help providers interested in programs that use health coaches or digital health programs to tailor inventions that increase patient care. Our key findings are as follows: (1) participants and clinicians valued the convenience of digital appointments; (2) health coaches provide tailored support to meet the needs of patients and parents; (3) coaching equips participants with key mental health skills such as positive thinking, improved communication, and proactive problem-solving; and (4) bilingual and bicultural health coaches can enhance accessibility and effectiveness of mental health services for Spanish-speaking patients. Our findings highlighting the convenience and value of digital mental health interventions are consistent with previous research reporting higher levels of satisfaction among patients [8-10]. Participants cited similar reasons for their preference for web-based services that other literature have highlighted, such as improved accessibility and increased flexibility [8,9].

Participants elaborated more about why they preferred the increased accessibility and flexibility digital mental health services offer. Parents in our study oftentimes balance competing demands such as having one or more jobs, so the flexibility of health coaches to virtually meet after standard business hours made it possible for parents to participate. Of the 11 parents, 36% (n=4) reported having a steady living situation at the time of the interview but worried about maintaining their situation. Therefore, similar programs might benefit from connecting with school officials and leaders, especially at schools with lower SES. Doing so could help families who most need the resources and support that programs like YFE offer. By partnering with school counselors and actively doing outreach in underserved communities, similar programs might better identify and support adolescents and families who may benefit from additional support.

Parents and patients come to mental health programs with a range of needs that may require different levels of support. Therefore, it is important to remain flexible and open when assessing parent and patient needs and determining mode of communication, program duration, and meeting interval frequency. Although video or in-person sessions may not work for every participant, other parents and adolescent patients might benefit from expanding their web-based meetings with health coaches. Embracing video platforms could facilitate the connection between participants and health coaches and increase rapport and engagement. This digital face-to-face interaction could be especially impactful for participants with transportation challenges or difficulty in establishing rapport over the phone. Additionally, participants might benefit from flexibility in program duration and the frequency of meeting intervals to better address parent and patient needs. Some families might necessitate longer-term support beyond the standard 6-month timeframe, while others might benefit from shorter-term support to meet their needs. Also, some families could benefit from meeting more frequently with health coaches beyond the standard monthly meeting for parents and biweekly meetings for adolescent patients.

In this study, we saw an opportunity for our program to further impact populations that may not have had access to mental health services before. Of our parent participants, 73% (8/11) identified as Hispanic and 64% (7/11) were Spanish speakers. Most of the Hispanic and Spanish-speaking parents in our program remarked on navigating the United States as immigrants, with 1 parent describing feeling “all alone” in this country. These sentiments are consistent with studies that have shown how stressors experienced by immigrants, like financial hardship and postmigration difficulties, are associated with mental illness and distress [23,24]. Most of Spanish-speaking parents in the YFE program reported feeling high levels of support from their health coach who spoke Spanish and had the cultural competency to understand their experiences as either Hispanic or an immigrant in the United States. These experiences underscore the pivotal role that bilingual health coaches play in providing culturally sensitive and effective support, which enhances the accessibility and effectiveness of mental health services for Spanish-speaking families. This finding is consistent with other research looking at immigrant populations in countries like Norway and Australia who benefited from mental health providers who shared patients’ cultural background and same language [25-27]. Those studies found that mental health coaches who shared a similar cultural background to patients better equipped them to understand cultural nuances, values, and beliefs which helped foster trust and rapport with patients and led to better communication and understanding [25-27]. Although the YFE program was not designed specifically for Spanish-speaking families, we found that our program was particularly impactful for this population since we had a Spanish-speaking health coach who shared the cultural background of Hispanic families participating in the program.

Both parents and patients cited the strong rapport they developed with their health coach, with 1 parent calling her health coach her “friend” and even her “angel.” Although there is literature that documents the high levels of support and alliance patients can establish with clinicians through digital methods, we were surprised to hear the highly positive language used to describe participants’ relationships with their health coach, especially by parents in our program [11,12]. Therefore, other programs could benefit by increasing behavioral health support for parents as well as adolescent patients. Cultivating more space for parents to honestly share their experiences may prove fruitful for other programs. Increased program content around behavioral health and emotional support could increase program effectiveness, especially for vulnerable populations. Although most of the current literature about digital health coaches specifically works with adult populations [13,14,17,28], this study demonstrates that interventions that work with adolescent patients and parents can facilitate a supportive environment where both feel heard and valued, ultimately enhancing their mental well-being and the parent-child relationship.

By implementing these suggestions for change, similar web-based mental health programs for adolescents and their patients can enhance accessibility, flexibility, and effectiveness, which could possibly improve outcomes and advance the well-being of young patients facing diverse mental health challenges.

### Limitations

The main limitation of our evaluation is selection bias. Our interview sample was composed of parents and patients actively involved in the YFE program and referred to the evaluation team by health coaches. These participants were more likely to have the time and motivation to participate in interviews to share their program experiences. Data collection occurred over the span of 3 months. The YFE program had been in operation for 18 months prior to interview recruitment commencing, with 62 patients having dropped out prior to our evaluation, primarily due to health coaches being unable to reach them for follow-up. Given that interview participation was limited to patients active in the program, we did not collect the experiences of patients and parents who had withdrawn. This may contribute to bias and yield more positive experiences of the YFE program. In spite of these limitations, our qualitative evaluation provided descriptive details of participants’ experiences with the YFE program. Further, since we collected data from patients, parents, and providers, we were able to triangulate our findings to identify the themes that were consistent across all participants, which informed our suggestions.

### Conclusions

Web-based health coach programs may play a key role in providing wraparound care to adolescent patients receiving mental health services and their families. Participants routinely cited satisfaction with the YFE program, mentioning specific benefits that could potentially be leveraged by future interventions, such as the convenience of web-based appointments, the importance of tailored support, improved parent-child communication, and the value of speaking with bilingual health coaches in their native languages. Furthermore, the significance of health coach–parent concordance, both pertaining to language and culture competency, was frequently noted as facilitating stronger relationships and may be important to promote both improved outcomes and sustained participant engagement in digital mental health programs. Future research should explore the impact of culture and language on participants’ relationships with mental health professionals, particularly in digital spaces, as these may be essential to providing safe, responsive, and culturally informed care.

## Appendix

Multimedia Appendix 1 Consolidated Criteria for Reporting Qualitative Research (COREQ) research.

Multimedia Appendix 2 Patient interview guide.

Multimedia Appendix 3 English parent interview guide.

Multimedia Appendix 4 Spanish parent interview guide.

Multimedia Appendix 5 Referrer interview guide.

## Abbreviations

COREQ: Consolidated Criteria for Reporting Qualitative Research
SES: socioeconomic status
YFE: Youth and Family Engagement

## Data Availability

The data sets generated during or analyzed during this study are available from the corresponding author on reasonable request.
