# Supplementary material for: Digital Youth and Family Engagement Program for Adolescents Who Receive Outpatient Mental Health Services: Qualitative Evaluation
Source: JMIR Form Res. 2024 Oct 31;8:e60317. doi: 10.2196/60317 (PMC11565079; doi:10.2196/60317)
Supplement: Multimedia Appendix 2 [file formative_v8i1e60317_app2.docx]

**Youth and Family Engagement (YFE) Program**

Patient Interview Guide

1. Patient experience
2. What has been your experience working with a health coach in the YFE program [Youth and Family Engagement]?
   - Generally speaking, what are some of the things that you work on with your health coach? [e.g., challenges with school or situations in your social life.]
3. What are the things in the YFE program that you feel work well? [This can be anything – care delivery, accessibility, scheduling, etc.]
   - Has your health coach shared any strategies or skills that you have found to be helpful? Like what?
4. What are some things in the YFE program that you think could be improved? How so?
5. How would you evaluate your relationship with your health coach? [Do you comfortable speaking with them? Are they helpful?]
   - Is there anything about your relationship with your health coach that you wish was different?
6. Thinking about the goals that you want[ed] to achieve from this program, how did you and your health coach go about deciding on these?
   - Did you find your health coach supportive of your ideas?
   - Have you ever discussed your program goals with anyone else, besides your health coach? [e.g., a teacher or counselor]
     - How did those conversations go? [Are they supportive?]
   - How successful would you say that you have been in achieving the goals that you set?
7. Do you feel that your health coach provides you all the support you need to meet your goals?
   - Are there any additional resources or support that you think would be helpful?
8. When meeting with your health coach, do you have any concerns about the privacy of any of the topics that you discuss? [Why or why not?]
   - Have you discussed privacy at all with your health coach or anyone else in the program? If so, how did that conversation go?
9. Patient barriers to enrollment and sustained engagement
10. Thinking back to when you first joined the program, how was that initial enrollment process for you?
    - Did you feel it was easy to get started in the program? [Why or why not?]
    - Was there anything about the initial enrollment process that could have made it easier for you to join the program?
11. When you first joined, what were you hoping to get out of the program?
12. In terms of your regular meetings with your health coach, do you have any challenges attending these? [e.g., scheduling a time to talk or finding a quiet space for the call]
    - Is there anything that could make attending these meetings easier for you?
13. Typically, participants remain enrolled in the program for 6 months, meeting with their health coaches twice a month. What do you think of that schedule?
    - Would you like to be contacted more often? Less often? [Why?]
    - Do you think 6 months is a good duration for the program? [Why or why not?]
14. Have you ever missed or had to reschedule an appointment?
    - *If so:* What caused you to miss your appointment?
      - What was the rescheduling process like for you?
      - Was there anything that could have made rescheduling easier?
15. Outside of regular appointments, do you feel like you are able to contact your health coach or other program staff, if you needed to? [Why or why not?]
16. Overall satisfaction and impact
17. Overall, is there anything about your communication with your health coach, or anyone else associated with the program, that you think could be improved? [In what ways?]
18. Is there anything about your participation in the program that you wish could be different? [How so?]
19. Is there anything that we did not discuss that you think would be important for us to consider as we evaluate this program?
