# Supplementary material for: Digital Youth and Family Engagement Program for Adolescents Who Receive Outpatient Mental Health Services: Qualitative Evaluation
Source: JMIR Form Res. 2024 Oct 31;8:e60317. doi: 10.2196/60317 (PMC11565079; doi:10.2196/60317)
Supplement: Multimedia Appendix 3 [file formative_v8i1e60317_app3.docx]

**Youth and Family Engagement (YFE) Program**

Parent/Guardian Interview Guide

1. Parent/Guardian experience
2. What has been your experience working with a health coach in the YFE program [Youth and Family Engagement]?
   - During your calls with the health coach, what are some of the things that you discuss? [e.g., concerns pertaining to your child or specific needs of the family.]
   - Do you feel that your time meeting with the health coach is helpful? [How so?]
3. Do you feel that your child has benefitted from their time in the YFE program? [How so?]
4. What are the things in the program that you feel work well? [This can be anything – care delivery, accessibility, scheduling, etc.]
   - Has the health coach shared any strategies or skills that either you or your child have found to be helpful? [Like what?]
5. Are there things about the YFE program that you think could be improved? [Like what?]
6. How would you evaluate your relationship with the health coach? [Do you comfortable speaking with them? Are they helpful?]
   - Is there anything about your relationship with the health coach that you wish was different?
   - How would you evaluate your child’s relationship with the health coach?
7. Thinking about both you and your child’s goals for this program, how did you, your child, and the health coach go about deciding on these?
   - Did you find the health coach supportive of your ideas? [Why or why not?]
   - Do you feel that both you and your child participated in setting these goals?
   - How successful would you say that your child has been in achieving the goals that were set?
8. Do you feel that the health coach provides you and your child with all the support that you both need to meet your goals?
   - Are there any additional resources or support that you think would be helpful?
9. When meeting with the health coach, do you feel that they do a good job keeping you up-to-date about your child’s progress in the program?
   - Is there any additional information that you’d want the health coach to provide?
10. Parent/Guardian barriers to sustained engagement
11. Thinking back to when your child first joined the program, what was that initial enrollment process like?
    - Did you feel it was easy to get started in the program? [Why or why not?]
    - Was there anything about the initial enrollment process that could have made it easier for you to enroll your child in the program?
12. When your child first joined, what were you hoping that they’d get out of the program?
    - Did you have any hesitations about your child joining the program? [Like what?]
    - When enrolling your child in the YFE program, did you have any specific goals for either yourself or your family? [e.g., access to community resources.]
13. In terms of your regular meetings with the health coach, do you have any challenges attending these? [e.g., scheduling a time or finding a quiet space for the phone call]
    - Is there anything that would make it easier for you to meet with the health coach?
14. Does your child have any challenges attending their meetings with the health coach? [Like what?]
    - Is there anything that would make it easier for them to meet the health coach?
15. Parents and guardians are typically contacted once a month, what are your thoughts on that time schedule?
    - Would you like to be contacted more often? Less often? [Why?]
16. Have you ever missed or had to reschedule an appointment?
    - *If so:* What caused you to miss your appointment?
      - What was the rescheduling process like for you?
      - Was there anything that could have made rescheduling easier?
17. If you needed to, do you feel like you are able to get in contact with the health coach or other program staff? [Why or why not?]
18. Overall satisfaction and impact
19. Do you find meeting with the health coach to be a valuable use of your time? [How so?]
    - Are there any specific aspects that have been most helpful for you, as a parent?
    - Are there any specific aspects that have been most helpful for your child?
20. Overall, is there anything about your communication with the health coach, or anyone else associated with the program, that you think could be improved? [In what ways?]
21. Is there anything about your participation in the program that you wish could be different? [How so?]
    - Is there anything that you wish could be different about your child’s participation in the program?
22. Is there anything that we did not discuss that you think would be important for us to consider as we evaluate this program?
