# Supplementary material for: Digital Youth and Family Engagement Program for Adolescents Who Receive Outpatient Mental Health Services: Qualitative Evaluation
Source: JMIR Form Res. 2024 Oct 31;8:e60317. doi: 10.2196/60317 (PMC11565079; doi:10.2196/60317)
Supplement: Multimedia Appendix 4 [file formative_v8i1e60317_app4.docx]

**Programa de participación de jóvenes y familias (YFE)**

Guía de entrevistas para padres/tutores

1. Experiencia de padres/tutores
2. ¿Cuál ha sido su experiencia trabajando con un entrenador de salud en el programa de jóvenes y familias?
   - Durante sus llamadas con el entrenador de salud, ¿cuáles son algunas de las cosas que conversan? [por ejemplo, inquietudes relacionadas con su hijo o necesidades específicas de la familia.]
   - ¿Siente que su tiempo de reunión con el entrenador de salud es útil? [¿Me puede decir más?]
3. ¿Siente que su hijo ha beneficiado de su tiempo en el programa? [¿Me puede decir más?]
4. ¿Cuáles son algunas cosas en el programa que siente que funcionan bien? [Esto puede ser cualquier cosa: los servicios de salud, la accesibilidad, la programación, etc.]
   - ¿El entrenador de salud ha compartido alguna estrategia o habilidad que usted o su hijo hayan encontrado útiles? [¿Cómo qué?]
5. ¿Hay cosas sobre el programa que podrían mejorarse? [¿Cómo qué?]
6. ¿Cómo evaluaría su relación con el entrenador de salud? [¿Se siente cómodo hablando con ellos? ¿Son útiles?]
   - ¿Hay algo en su relación con el entrenador de salud que desearía que fuera diferente?
   - ¿Cómo evaluaría la relación de su hijo con el entrenador de salud?
7. Pensando en las metas de usted y de su hijo para este programa, ¿cómo decidieron usted, su hijo y el entrenador de salud acerca de esto?
   - ¿Encontró que el entrenador de salud apoyaba sus ideas? [¿Por qué o por qué no?]
   - ¿Siente que tanto usted como su hijo participaron en el establecimiento de estas metas?
   - ¿Qué tan exitoso diría que ha sido su hijo en lograr las metas que se establecieron?
8. ¿Siente que el entrenador de salud le brinda a usted y a su hijo todo el apoyo que ambos necesitan para alcanzar sus metas?
   - ¿Hay algún recurso o apoyo adicional que sería útil?
9. Al reunirse con el entrenador de salud, ¿siente que hace un buen trabajo al mantenerlo actualizado sobre el progreso de su hijo en el programa?
   - ¿Hay alguna información adicional que le gustaría que el entrenador de salud le proporcione?
10. Barreras para la participación sostenida de los padres/tutores
11. Pensando en cuando su hijo se unió al programa por primera vez, ¿cómo fue el proceso de inscripción?
    - ¿Sintió que fue fácil iniciarse en el programa? [¿Por qué o por qué no?]
    - ¿Hubo algo en el proceso de inscripción inicial que podría mejorar o haber hecho el proceso de inscripción más fácil?
12. Cuando su hijo se unió por primera vez al programa, ¿qué esperaba que obtuviera del programa?
    - ¿Tuvo alguna duda acerca de que su hijo se uniera al programa? [¿Cómo qué?]
    - Al inscribir a su hijo en el programa, ¿tenía metas específicas para usted o su familia? [por ejemplo, acceso a los recursos de la comunidad.]
13. Con respecto a sus reuniones periódicas con el entrenador de salud, ¿tiene algún desafío para asistir a estas reuniones? [por ejemplo, alguna dificultad en programando una hora o encontrando un lugar tranquilo para la llamada telefónica]
    - ¿Hay algo que podría hacer más fácil reunirse con el entrenador de salud?
14. ¿Su hijo tiene algún desafío para asistir a sus reuniones con el entrenador de salud? [¿Cómo qué?]
    - ¿Hay algo que podría hacer más fácil a su hijo en reunirse con el entrenador de salud?
15. Por lo general, se contacta a los padres y tutores una vez al mes, ¿cuál es su opinión sobre ese horario?
    - ¿Le gustaría ser contactado más a menudo? ¿Con menos frecuencia? [¿Por qué?]
16. ¿Alguna vez ha perdido o ha tenido que reprogramar una cita?
    - *En ese caso:* ¿Qué le hizo faltar a su cita?
      - ¿Cómo fue para ti el proceso de reprogramar una cita?
      - ¿Había algo que podría hacer más fácil el proceso de reprogramar una cita?
17. Si lo necesitara, ¿siente que puede ponerse en contacto con el entrenador de salud u otro personal del programa? [¿Por qué o por qué no?]
18. Satisfacción general e impacto
19. ¿Considera que reunirse con el entrenador de salud es un uso valioso de su tiempo? [¿Cómo es eso?]
    - ¿Hay algún aspecto específico que haya sido más útil para usted como padre?
    - ¿Hay algún aspecto específico que haya sido más útil para su hijo?
20. En general, ¿hay algo sobre su comunicación con el entrenador de salud o cualquier otra persona asociada con el programa que crea que podría mejorarse? [¿De qué manera?]
21. ¿Hay algo acerca de su participación en el programa que le gustaría que fuera diferente? [¿Cómo es eso?]
    - ¿Hay algo que le gustaría que fuera diferente en la participación de su hijo en el programa?
22. ¿Hay algo que no discutimos que usted cree que sería importante que consideráramos en nuestra evaluación de este programa?
