# Supplementary material for: Digital Youth and Family Engagement Program for Adolescents Who Receive Outpatient Mental Health Services: Qualitative Evaluation
Source: JMIR Form Res. 2024 Oct 31;8:e60317. doi: 10.2196/60317 (PMC11565079; doi:10.2196/60317)
Supplement: Multimedia Appendix 5 [file formative_v8i1e60317_app5.docx]

**Youth and Family Engagement (YFE) Program**

Referrer Interview Guide

1. Identifying and referring potential participants
2. What has been your experience referring adolescents to the YFE program [Youth and Family Engagement]?
   - Do you experience any challenges referring potential participants to the YFE program? [If so, how do you navigate these challenges?]
   - What about the referral process could be improved?
   - What about the referral process works well?
3. What is your process for deciding which adolescents and their families might be good candidates for the YFE program? [Do you have a set criteria or certain things that you most look for?]

- Do you have any exclusion or rule out criteria when it comes to referring potential participants? [e.g., not attending counseling appointments, medication nonadherence]

1. Once you have identified a potential participant, how do you go about informing them of the YFE program?

- What does your communication with the family look like?
- Do you have any resources or handouts that you share with adolescents and their families?
- Other than yourself, is anyone else involved in the referral process or initial family discussion? [e.g. school staff, teachers, other care team members]

1. What has been your experience working with families that may not speak English?
   - What languages have you encountered in the referral process so far?
2. How do you navigate potential language barriers for families that may not speak English?
3. What about the referral process could be improved to better support families that may not speak English? [e.g., additional staff, access to interpreters, translated resources, etc.]
4. When first discussing the possibility of the Y**F**E program with adolescents and their families, what is their general response?
   - Are there parts of the program that seem to be most appealing to adolescents and their families? [Like what?]
   - Do you notice any commonly occurring concerns among adolescents or their families? [e.g., time commitment, language barriers]
   - As rough estimate, what would you say is the breakdown of adolescents and families that choose to be referred to the YFE program and those that don’t?
5. For adolescents and families that decide to be referred to the YFE program, what do you think that they hope to get from the program? [Are there any specific goals?]
6. From your observation, do adolescents and families face any challenges navigating the referral process? [e.g., trouble connecting with YFE staff, language barriers]

- How do they navigate these challenges?
- Do you ever assist them with the referral process?

1. Contact with YFE participants post program enrollment
2. Once an adolescent is enrolled in the YFE program, do you ever discuss their experiences while in the program?

If yes, continue to Question 8.

If no, skip to Question 10.

1. Generally speaking, do you think the YFE program is helpful for adolescents and their families? [In what ways?]
   - Are there specific things about the program that you feel work well?
   - Are there specific things about the program that you think could be improved?
2. Is there any additional assistance or resources that you think the program could provide to adolescents and their families, that it is not currently providing?
   - How would this assistance or these resources be helpful?
3. Typically, participants remain enrolled in the program for 6 months, meeting with their health coaches twice a month. What do you think of that schedule?
   - Do you think adolescents should be contacted more often? Less often? [Why?]
   - Parents and/or caregivers are contacted by health coaches once a month, what do you think of that time interval?
   - Do you think 6 months is a good duration for the program? [Why or why not?]
4. Outside of referring potential participants, do you ever have contact with YFE health coaches or other YFE staff?
   - If so, what do those conversations entail?
   - Do you think it would be beneficial for YFE health coaches to have direct contact with participants’ counselors or their psychiatric care team? [Why?]
     - If so, is there any specific information that you think would be most beneficial to share?
5. Overall satisfaction and impact
6. Overall, how successful do you think the YFE program is at helping adolescents and their families?
   - Are there any specific areas in which you think the program does really well?
7. Is there anything about adolescents’ participation in the YFE program that you think could be improved? [Like what?]
8. Is there anything that we did not discuss that you think would be important for us to consider as we evaluate this program?
